# Diabetic Foot Osteomyelitis Undergoing Amputation: Epidemiology and Prognostic Factors for Treatment Failure

**DOI:** 10.1093/ofid/ofae236

**Published:** 2024-07-09

**Authors:** Yae Jee Baek, Eunjung Lee, Jongtak Jung, Sung Hun Won, Chi Young An, Eun Myeong Kang, Se Yoon Park, Seung Lim Baek, Dong-il Chun, Tae Hyong Kim

**Affiliations:** Division of Infectious Diseases, Department of Internal Medicine, Soonchunhyang University Seoul Hospital, Soonchunhyang University College of Medicine, Seoul, Republic of Korea; Division of Infectious Diseases, Department of Internal Medicine, Soonchunhyang University Seoul Hospital, Soonchunhyang University College of Medicine, Seoul, Republic of Korea; Division of Infectious Diseases, Department of Internal Medicine, Soonchunhyang University Seoul Hospital, Soonchunhyang University College of Medicine, Seoul, Republic of Korea; Department of Orthopedic Surgery, Soonchunhyang University Seoul Hospital, Soonchunhyang University College of Medicine, Seoul, Republic of Korea; Department of Orthopedic Surgery, Soonchunhyang University Seoul Hospital, Soonchunhyang University College of Medicine, Seoul, Republic of Korea; Department of Orthopedic Surgery, Soonchunhyang University Seoul Hospital, Soonchunhyang University College of Medicine, Seoul, Republic of Korea; Division of Infectious Diseases, Department of Internal Medicine, Hanyang University Seoul Hospital, Seoul, Republic of Korea; Department of Orthopedic Surgery, Soonchunhyang University Seoul Hospital, Soonchunhyang University College of Medicine, Seoul, Republic of Korea; Department of Orthopedic Surgery, Soonchunhyang University Seoul Hospital, Soonchunhyang University College of Medicine, Seoul, Republic of Korea; Division of Infectious Diseases, Department of Internal Medicine, Soonchunhyang University Seoul Hospital, Soonchunhyang University College of Medicine, Seoul, Republic of Korea

**Keywords:** antimicrobial stewardship, diabetic foot infection, lower extremity amputation, multidisciplinary approach, osteomyelitis

## Abstract

**Background:**

When treating diabetic foot osteomyelitis (DFO), it remains difficult to determine the presence of residual infection and the optimal treatment after bone resection. In this study, we aimed to investigate the clinical characteristics of and prognostic factors in patients with DFO undergoing amputation.

**Methods:**

This retrospective study involved 101 patients with DFO who underwent amputation. Data on their demographics, clinical characteristics, tissue culture, and surgery type were collected. Patients were grouped according to primary closure status and clinical outcome postamputation. A good outcome was defined as a successful complete remission, characterized by the maintenance of complete wound healing with no sign of infection at 6 months postamputation. Multivariate logistic regression analysis was performed. Outcomes according to surgery type were also analyzed.

**Results:**

*Staphylococcus aureus* (17%) and *Pseudomonas* species (14%) were the most prevalent pathogens. Gram-negative bacteria were isolated from 62% of patients. In patients with primary closure, hemodialysis and ankle brachial index (ABI) <0.6 were associated with poor outcomes. In patients with DFO, ABI <0.6 was the only prognostic factor associated with treatment failure. Antimicrobial stewardship allows patients who underwent major amputation to reduce the duration of antibiotic therapy compared to those after minor amputation, although it did not contribute to reducing mortality.

**Conclusions:**

Peripheral artery disease and hemodialysis were associated with poor outcomes despite radical resection of the infected bone. Vigilant monitoring after amputation and antimicrobial stewardship implemented based on microbiological epidemiology, prognostic factors, and the type of surgery are important. A multidisciplinary team could assist in these activities to ensure treatment success.

## Teaser

Peripheral artery disease and hemodialysis were associated with poor outcomes despite radical resection of the infected bone. Vigilant monitoring after amputation and antimicrobial stewardship are important. A multidisciplinary team could assist in these activities to ensure treatment success.

More than 15% of patients with diabetic foot infection (DFI) die within a year of diagnosis, and 17% undergo major lower extremity amputation (LEA) [1]. Diabetic foot osteomyelitis (DFO) is usually caused by the spread of a soft tissue infection from nonhealing ulcers to the bone, with approximately half of the patients presenting severe infections [2]. DFO is associated with fatal outcomes, including LEA. As antibiotics cannot reach the bone cortex, a longer duration of antibiotic treatment is usually required for DFO. Amputation is typically indicated when the infection cannot be controlled, the microorganism is difficult to treat or does not respond to medical treatment, or the infection site is extensive and necrotic. As primary surgical closure after amputation is not associated with complications such as exudation, edema, and reinfection [3], it is usually performed with radical resection. However, primary closure cannot be performed in some cases of residual infection. Postoperative antibiotic therapy is recommended based on the status of the stump site [4]. If radical resection is performed without residual infected tissue, a short treatment duration (2–5 days) is proposed; prolonged treatment is suggested if there are residual infected tissues. Specifically, prolonged administration of antibiotic therapy is considered when the infection is extensive and the patient has severe peripheral artery disease (PAD) [5]. Bone margin cultures are recommended to determine the type and duration of antimicrobial treatment needed [6].

According to the International Working Group on the Diabetic Foot/Infectious Diseases Society of America guidelines on the diagnosis and treatment of diabetes-related foot infections [7], information on how to determine the optimal treatment duration for DFO is limited, and it remains difficult to determine the presence of residual infection after bone resection, which is usually decided by someone with clinical expertise. As the extended use of broad-spectrum antibiotics is not beneficial to patients, a multidisciplinary team should be involved in comprehensive patient assessment, and antimicrobial stewardship should be implemented to achieve optimal outcomes. Therefore, in this study, we aimed to investigate the clinical characteristics, including the causative organisms, prognostic factors, and clinical outcomes, of patients with DFO who underwent amputation in the clinical setting using a regular multidisciplinary approach.

## METHODS

### Study Design

This retrospective cohort study was performed in a 750-bed teaching hospital in the Republic of Korea. All consecutive hospitalized patients with type 2 diabetes mellitus diagnosed with DFO between December 2017 and May 2022 were screened for inclusion in the cohort. Among them, 101 patients who underwent an amputation were included. DFO was assessed using 3 types of imaging: magnetic resonance imaging, white blood cell single-photon emission computed tomography, and 3-phase bone scan. Additionally, these findings were confirmed via bone culture and histopathology. Data on demographics, underlying conditions, initial DFI, peripheral vascular status, tissue culture, and surgical history were collected. After LEA, the progress and prognosis of DFO treatment were also obtained.

The study cohort was divided into 2 groups according to the presence of primary closure (PC). PC denotes the radical resection of infected tissues. Orthopedic surgeons performed PC if they assessed the amputation site as clean. Bone margin culture was conducted by the surgeon's decision. Residual infection (RI) refers to the nonachievement of PC because of the presence of residual infected tissue despite LEA. In such instances, the wound is typically left open and treated with vacuum-assisted closure. During this retrospective study, 2 orthopedic surgeons conducted a comprehensive review by examining the medical charts and surgical photographs of the patients to evaluate the presence of RI.

**Figure 1.**
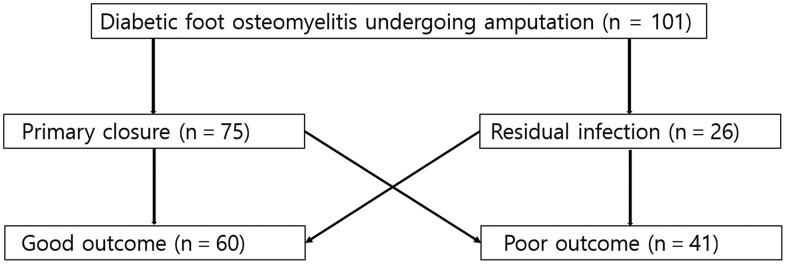
Study design and population. The cohort was divided into 2 groups according to the presence or absence of primary closure (primary closure and residual infection). Primary closure and residual infection denote radical resection of infected tissue and the presence of residual infected tissue, respectively. The groups were also subcategorized as having good or poor outcomes. A good outcome was defined as successful complete remission, which is the maintenance of a clean wound for 6 months after amputation with complete healing of the wound and no sign of infection. Otherwise, the patients were classified as having a poor outcome.

PC and RI were additionally subcategorized into good and poor outcomes (GOs and POs, respectively), forming a total of 4 groups. Outcomes were determined by the achievement of remission after amputation. A GO was defined as a successful complete remission, characterized by the maintenance of complete wound healing with no sign of infection at 6 months postamputation. Otherwise, the patient was classified as having a PO. Therefore, the study population was divided into the following groups: PC-GO, PC-PO, RI-GO, and RI-PO. The scheme of this study is shown in Figure 1.

Risk factors for a PO in patients with DFO after amputation were analyzed. We also collected data pertaining to the need for additional amputation, duration of antibiotic use, antibiotic-related adverse events (including *Clostridioides difficile* infection [CDI]), presence of newly acquired multidrug-resistant organisms (MDROs), and in-hospital mortality. MDROs include carbapenem-resistant Enterobacterales, vancomycin-resistant *Enterococcus*, and other nonfermenting gram-negative bacteria resistant to at least 1 agent in 3 or more antimicrobial categories. Last, the prognosis was analyzed according to surgery type: major or minor amputation. Major amputation refers to below-knee amputation, whereas minor amputation refers to ray or transmetatarsal amputation.

### Multidisciplinary Approach and Antimicrobial Stewardship

All patients with DFI were hospitalized and evaluated by a multidisciplinary team consisting of orthopedic surgeons, infectious disease specialists, endocrinologists, interventional radiologists, reconstructive surgeons, cardiovascular surgeons, and rehabilitation specialists. The team held weekly meetings hosted by 2 orthopedic surgeons to improve communication and allow rapid decision-making to minimize pedal complications in hospitalized patients with DFI. Percutaneous transluminal angiography or bypass surgery was performed for patients with PAD. The surgical sites were reviewed and discussed to determine the appropriate medical treatment or surgical reconstruction after amputation. Simultaneously, antimicrobial stewardship and infection control measures were implemented. Antimicrobial stewardship involves the optimal selection and dose of empirical and definite antibiotics as well as the duration of antibiotic use according to the clinical syndrome. Empirical and targeted antibiotics were recommended and the period of antibiotic treatment was discussed every week. Specifically, when radical resection is performed without residual infected tissue, a short treatment duration within 5 days is proposed. If the residual infected part involves soft tissue after amputation, 1–3 weeks of antimicrobial treatment is proposed; 4–6 weeks of treatment is proposed if the bone is involved [5]. The patients were referred for rehabilitation after surgery and during prolonged hospitalization.

### Identification of Causative Pathogens, PAD, and Ankle Brachial Index

Causative pathogens were identified via tissue culture after amputation surgery. Cultures of the bones or deep tissues, which were obtained from careful debridement before or during amputation, were included. The pathogens, adequacy of antibiotics, and duration of antibiotic treatment were confirmed and reviewed by infectious disease specialists.

All diabetic foot lesions were graded according to the PEDIS (perfusion, extent/size, depth/tissue loss, infection, and sensation) system, a classification system developed by the Infectious Diseases Society of America [5]. Grade 1 denotes superficial infection with localized cellulitis (≤2 cm); grade 2 denotes moderate infection extending to the joint, bone, or abscess; grade 3 represents severe infection; and grade 4 indicates local infection with a systemic inflammatory response. PAD is defined as the presence of at least 1 segment with more than 50% stenosis or occlusion of the diabetic foot site on computed tomography angiography [8]. The ankle brachial index (ABI) is another diagnostic tool that facilitates PAD diagnosis. An ABI of <0.9 indicates the presence of PAD [9]. In our study, PAD was diagnosed using computed tomography angiography; the ABI was also determined.

### Statistical Analyses

Continuous variables are presented as the mean and standard deviation or median and interquartile range. Categorical variables are presented as numbers and percentages. The χ^2^ and Fisher exact tests were used for univariate comparisons of categorical data. Variables that were not normally distributed were analyzed using the Mann-Whitney *U* test. The Kruskal-Wallis test was used to compare 3 or more groups of variables. Variables with *P* < .2 in the univariate analysis were included in the multivariate logistic regression analysis. Adjusted odds ratios (ORs) and 95% confidence intervals (CIs) were also analyzed for POs in patients with DFO undergoing amputation and PC. Owing to the collinearity between variables, we included the ABI classification and end-stage renal disease (ESRD) in the multivariate logistic regression analysis as replacements for PAD and chronic kidney disease, respectively. All *P* values were 2-tailed, and *P* < .05 was considered statistically significant. Statistical analyses were performed using R Studio software (version 4.2.1).

## RESULTS

### Characteristics of the Study Population

The mean age of the overall study population was 67.5 years, and there were 60 male patients (59.4%). Medical aid was provided to 28.7% of the patients; 88.9% of the patients had diabetes for >10 years. Forty (40.6%) patients with ESRD were on hemodialysis; none were on peritoneal dialysis. Thirty-eight patients (37.6%) had a history of DFI before a DFO episode. The demographic and baseline characteristics of the patients are described in Table 1.

**Table 1.** Baseline Characteristics of the 4 Groups in the Study Population

Characteristic	Primary Closure^a^			Residual Infection^a^		
	Good Outcome^b^ (n = 49)	Poor Outcome^b^ (n = 26)	*P* Value	Good Outcome^b^ (n = 11)	Poor Outcome^b^ (n = 15)	*P* Value
Age, y, median (IQR)	70 (63–79)	66.5 (58–73.5)	.26	63 (58.5–70)	69 (52.5–73.5)	.88
Sex, male	27 (55.1)	14 (53.8)	1	7 (63.6)	12 (80.0)	.41
Body mass index, kg/m^2^, median (IQR)	23.2 (21.3–26.0)	23.5 (21.3–25.0)	.94	26.5 (20.9–27.2)	23.4 (21.0–26.5)	.72
Medical aid	13 (26.5)	9 (34.6)	.64	2 (18.2)	5 (33.3)	.66
Smoker^c^	21 (42.9)	14 (53.8)	.51	3 (27.3)	6 (40.0)	.68
HbA1c, %, median (IQR)^d^	7.1 (6.3–8.4)	7.3 (6.5–8.9)	.53	7.9 (6.8–9.3)	7.7 (6.4–9.1)	.82
Diagnosis of DM over 10 y	43 (87.8)	23 (92)	.87	10 (100)	12 (80)	.25
Medical conditions						
CAOD	22 (44.9)	16 (61.5)	.26	6 (54.5)	6 (40.0)	.74
PAD	34 (70.28)	24 (92.3)	.06	7 (63.6)	13 (86.7)	.35
CKD	33 (71.7)	23 (92.0)	.09	8 (72.7)	9 (60.0)	.68
ESRD	23 (47.9)	19 (76.0)	.04	7 (63.6)	9 (60.0)	1
Dementia	8 (16.3)	1 (3.8)	.15	0 (0)	1 (6.7)	1
Malignancy	4 (8.2)	1 (3.8)	.65	0 (0)	0 (0)	ns
Osteoporosis	14/31 (45.2)	11/16 (68.8)	.22	1/7 (14.3)	2/10 (20)	1
History of DFI	20 (40.8)	15 (58.7)	.25	0 (0)	3 (20)	.24
Symptom onset to amputation, d, median (IQR)	58 (34–130)	67.5 (22.5–181.3)	.90	37 (24.5–42)	60 (23–103)	.27
Infection site			1			.12
Forefoot	33 (67.3)	18 (69.2)		6 (54.5)	12 (80.0)	
Midfoot	9 (18.4)	3 (11.5)		3 (27.3)	3 (20.0)	
Hindfoot	7 (14.3)	5 (19.2)		2 (18.2)	0 (0.0)	
PEDIS classification			.99			.61
Grade 2	5 (10.2)	0 (0)		0 (0)	1 (6.7)	
Grade 3	34 (69.4)	23 (88.5)		9 (81.8)	9 (60.0)	
Grade 4	10 (20.4)	3 (11.5)		2 (18.2)	5 (33.3)	
ABI			.03			.05
>0.8	25 (55.6)	6 (26.1)		7 (70)	4 (30.8)	
0.6–0.79	5 (11.1)	3 (13.0)		2 (20)	2 (15.4)	
0.4–0.59	3 (6.7)	4 (17.4)		0 (0)	5 (38.5)	
<0.39	12 (26.7)	10 (43.5)		1 (10)	2 (15.4)	
Multiple ulcers	17 (34.7)	12 (46.2)	.47	4 (36.4)	2 (13.3)	.35
Surgery type			.14			1
Major	19 (38.8)	5 (19.2)		1 (9.1)	2 (13.3)	
Minor	30 (61.2)	21 (80.8)		10 (90.9)	13 (86.7)	

Data are presented as No. (%) unless otherwise indicated.

Abbreviations: ABI, ankle brachial index; CAOD, coronary artery occlusive disease; CKD, chronic kidney disease; DFI, diabetic foot infection; DM, diabetes mellitus; ESRD, end-stage renal disease requiring hemodialysis; HbA1c, glycated hemoglobin; IQR, interquartile range; ns, not significant; PAD, peripheral artery disease; PEDIS, perfusion, extent/size, depth/tissue loss, infection, and sensation.

^a^Primary closure denotes radical resection of the infected tissue. Residual infection refers to nonachievement of primary closure because of the presence of residual infected tissue despite lower extremity amputation.

^b^A good outcome was defined as successful complete remission. Otherwise, the patient was classified as having a poor outcome.

^c^Smoker includes current smokers and ex-smokers.

^d^HbA1c level is expressed as %.

The age, sex, body mass index, medical aid, smoking history, initial glucose control level, and diabetes prevalence were comparable between patients in the PC-GO group and those in the PC-PO group. However, the proportion of patients with ESRD was higher in the PC-PO group than in the PC-GO group (76% vs 47.9%, *P* = .04). In addition, the proportion of patients with ABI <0.6 was higher in the PC-PO than the PC-GO group (PC-PO vs PC-GO: 60.9% vs 33.4%, *P* = .03). Most variables were comparable in the RI-GO and RI-PO groups. The RI-PO group also had a higher proportion of patients with ABI <0.6 than the RI-GO group despite having no statistically significant difference (RI-PO vs RI-GO: 53.9% vs 10%, *P* = .05).

### Causative Pathogens of DFO in Patients Undergoing Amputation

Bone culture during amputation was performed in 94 of 101 cases (93.1%). Among them, 28 showed no growth in bone tissue culture. However, 25 of 28 cases resulted in the presence of bacteria in tissue culture before amputation, which represents pathogens. All isolates obtained from clinically relevant samples were included (n = 119) and analyzed. *Staphylococcus aureus* accounted for 17% of all cases (methicillin-resistant *S aureus*, 7.6%), followed by *Pseudomonas* spp (14%), *Klebsiella* spp (11%), and *Escherichia coli* (10%). Gram-negative bacteria were isolated from 62% (76/122) of patients. The specific species or genera of the identified microorganisms are described in Table 2. Polymicrobial infections accounted for 31.5% of patients with DFO.

**Table 2.** Microbial Epidemiology of Diabetic Foot Osteomyelitis Requiring Amputation

Microorganism	No. (%) of Isolates
Gram-positive bacteria	
* Staphylococcus aureus*	20 (16.8)
* *Other staphylococci	11 (9.2)
* Streptococcus* sp	5 (4.2)
* Enterococcus* sp	10 (8.4)
* Pseudomonas* sp	16 (13.4)
Gram-negative bacteria	
* *Enterobacterales	
* Klebsiella* sp	13 (10.7)
* Escherichia coli*	12 (9.8)
* Enterobacter* sp	10 (8.2)
* Citrobacter* sp	9 (7.4)
* Proteus* sp	4 (3.2)
* Serratia* sp	4 (3.2)
* Morganella* sp	2 (1.6)
* *Nonfermenting gram-negative bacteria	
* Pseudomonas* sp	16 (13.1)
* Stenotrophomonas* sp	3 (2.5)
* Burkholderia* sp	1 (0.8)
* Acinetobacter* sp	2 (1.6)
Total	76 (62)

### Prognostic Factors of POs in Patients With DFO Undergoing Amputation

Patients with GOs did not undergo additional amputation, whereas 53.8% of patients in the PC-PO group and 93.3% of patients in the RI-PO group underwent additional LEA. Multivariate analysis was performed using the data from the PC group (Table 3). In the PC-GO and PC-PO groups, factors associated with POs were ABI <0.6 and ESRD. Specifically, patients with ABI values of 0.4–0.59 showed an OR of 12.6 (95% CI, 1.80–111.7; *P* = .014). Those with ABI <0.39 showed an OR of 6.50 (95% CI, 1.61–31.9; *P* = .013). Hemodialysis had an OR of 4.96 (95% CI, 1.38–22.5; *P* = .02).

**Table 3.** Factors Associated With Poor Outcomes in Diabetic Foot Osteomyelitis Patients Undergoing Amputation and Primary Closure

Factor	Crude OR (95% CI)	Adjusted OR (95% CI)	*P* Value
Major amputation	0.38 (.11–1.11)	0.30 (.07–1.15)	.10
ABI			
* *>0.8	1.00	1.00	
* *0.6–0.79	2.50 (.42–3.5)	2.74 (.42–16.7)	.27
* *0.4–0.59	5.56 (.99–35.4)	12.6 (1.80–111.7)	.014
* *<0.39	3.47 (1.05–12.5)	6.50 (1.61–31.9)	.013
ESRD	3.47 (1.24–10.9)	4.96 (1.38–22.5)	.02
Dementia	4.88 (.82–93.3)	2.72 (.34–59.6)	.41

Abbreviations: ABI, ankle brachial index; CI, confidence interval; ESRD, end-stage renal disease on hemodialysis; OR, odds ratio.

Factors affecting overall POs in patients with DFO after amputation were also analyzed (Supplementary Materials: Additional File 1). Although patients with RI after LEA had a higher PO rate than those with PC, the difference was not significant (OR, 2.96 [95% CI, .94–9.87]; *P* = .06). ABI <0.6 was the only factor associated with a poor prognosis (ABI 0.4–0.59: OR, 15.3 [95% CI, 3.1–102.7]; ABI <0.4: OR, 4.48 [95% CI, 1.34–16.4]).

### Additional Outcomes in Patients With DFO Undergoing Amputation

The median duration of antibiotic use after LEA was 44 and 50 days in the RI-GO and RI-PO groups, respectively. Conversely, the median duration of antibiotic use was shorter in the PC groups (15 days in the PC-GO group and 29.5 days in the PC-PO group). The incidences of CDI and MDRO were not significantly different between the PC and RI groups (CDI: PC, 12% vs RI, 23%, *P* = .20; MDRO: PC, 13% vs RI, 11%, *P* = 1.0) (Supplementary Materials: Additional File 2).

### Comparison of Patients Undergoing Major and Minor LEA

Table 4 describes the clinical characteristics and outcomes of patients with DFO in the major and minor amputation groups. The rate of PEDIS classification grade 4 in the major amputation group was higher than that in the minor amputation group, indicating that DFI was more severe in the major amputation group (major vs minor: 37% vs 13.5%, *P* = .04). After LEA, the period of antibiotic use following amputation was significantly shorter in the major group than in the minor group (major vs minor: 12 vs 31 days, *P* < .001); the need for additional amputation following LEA was also significantly lower in the major group than in the minor group (11.1% vs 33.8%, respectively; *P* = .02).

**Table 4.** Clinical Characteristics and Outcomes of Patients With Diabetic Foot Osteomyelitis According to the Extent of Their Amputation

Characteristic	Major Amputation (n = 27)	Minor Amputation (n = 74)	*P* Value
Age, y, median (IQR)	66 (64–74)	69 (58–79)	.94
Sex, male	14 (51.9)	46 (62.3)	.37
Body mass index, kg/m^2^, median (IQR)	22.7 (20.5–26.5)	23.4 (21.5–26.5)	.67
Medical aid	6 (22.2)	23 (31.1)	.46
Smoker^a^	12 (44.4)	30 (42.6)	1
Medical condition			
* *Coronary artery occlusive disease	15 (55.6)	35 (47.3)	.51
* *Peripheral artery disease	22 (88)	54 (75.0)	.26
* *Chronic kidney disease	18 (72.0)	55 (76.4)	.79
PEDIS classification			.04
* *Grade 2	2 (7.4)	4 (5.4)	
* *Grade 3	15 (55.6)	60 (81.1)	
* *Grade 4	10 (37.0)	10 (13.5)	
ABI			.07
* *>0.8	8 (33.3)	34 (50.7)	
* *0.6–0.79	4 (16.7)	8 (11.9)	
* *0.4–0.59	1 (4.2)	11 (16.4)	
* *<0.39	11 (45.8)	14 (20.9)	
Infection site			<.001
* *Forefoot	6 (22.2)	63 (85.1)	
* *Midfoot	8 (29.6)	10 (13.5)	
* *Hindfoot	13 (48.1)	1 (1.4)	
Severity			
* *Multiple ulcer	14 (51.9)	21 (28.4)	.03
* *Residual infection	3 (11.1)	23 (31.1)	.06
* *Duration of antibiotic therapy, d, median (IQR)	12 (7–21)	31 (14–50)	<.001
* *Complete remission	20 (74.1)	40 (58.8)	.10
* *Debridement	8 (29.6)	38 (51.4)	.07
* *Amputation	3 (11.1)	25 (33.8)	.02
* *CDI	5 (19.2)	9 (12.9)	.51
* *MDRO infection/colonization	5 (20)	8 (11.4)	.32
* *In-hospital mortality	5 (29.4)	6 (11.8)	.12

Data are presented as No. (%) unless otherwise indicated.

Abbreviations: ABI, ankle brachial index; CDI, *Clostridioides difficile* infection; IQR, interquartile range; MDRO, multidrug-resistant organism; PEDIS, perfusion, extent/size, depth/tissue loss, infection, and sensation.

^a^Smoker includes current smokers and ex-smokers.

## DISCUSSION

LEA is an undesirable outcome of DFI associated with a higher risk of death because of cardiovascular disease [10]. Amputation rates vary between centers and countries [11], and studies analyzing factors requiring LEA have been widely performed. Current independent predictors of LEA include osteomyelitis, ABI <0.9, uncontrolled glucose levels, ulcers for >1 month, and neuropathy [12–14]., Parameters to assess the prognosis after the surgical management of DFO include the presence of ischemia, necrosis, or soft tissue infection [15]. In our study, PAD, particularly ABI <0.6, was significantly associated with a poor prognosis of DFO after amputation. Considering the difficulty in interpreting computed tomography angiography results unless conventional angiography is performed, determining the ABI can assist in predicting outcomes. Hemodialysis was an independent factor associated with poor outcomes in patients who underwent radical resection. High levels of creatinine (≥1.3 mg/dL) and transfemoral amputation have been associated with high mortality after major amputation [16]. In addition, ESRD significantly increases the risk of major amputation after limb salvage with free flap [17]. Chronic kidney disease negatively affects wound healing by disrupting the keratinization process and reducing the rate of revascularization and cell proliferation [18]. Calcific arteriopathy, or calciphylaxis, is a serious condition associated with ESRD and the inevitable loss of proteins and soluble vitamins through renal replacement therapy [19]. Therefore, it could be inferred that ESRD could adversely affect the treatment of DFO. Consequently, it is recommended to approach patients with ESRD or severe ischemia with caution to prevent disease aggravation after LEA with radical resection.

In our study, patients who underwent initial major amputation were more likely to receive antibiotics for a shorter period, with a lower risk of additional surgery. Although we did not demonstrate a lower incidence of antibiotic-related complications and mortality in this study, this was noticeable considering the patients' comorbidities, the severity of DFI, and previous exposure to antibiotics. Therefore, major amputation in select patients with high-risk features could be associated with lower clinical failure rates and reduced antibiotic overuse. However, continued research to improve prognostic markers for clinical outcomes is important to optimize the balance between the twin goals of limb salvage and antimicrobial stewardship.

As the prevalence of antimicrobial resistance and consequent clinical deterioration has increased [20], antimicrobial stewardship in DFI has been recognized [21]. Antimicrobial stewardship in DFI remains challenging owing to the current trend of prolonged antibiotic use and the low rate of obtaining adequate samples. The multidisciplinary approach could assist in antimicrobial stewardship by considering poor prognostic factors and the presence of RI to determine the type and duration of antimicrobial treatment needed. In addition, evaluating the epidemiology of microorganisms in each community and hospital is important. Tissue cultures obtained from relevant samples showed that gram-negative bacteria were found in more than 60% of samples, indicating that empirical antibiotic coverage of gram-negative pathogens should be considered for treating patients with DFO. However, our findings cannot be generalized to institutions with different micro-epidemiological backgrounds. A similar study reported that 80% of patients had gram-positive bacteria and 42% had gram-negative bacteria [22]. In another study, 12.4% of cultures tested positive for methicillin-resistant *S aureus* and 17.5% tested positive for resistant gram-negative bacteria. Among these patients, 65% showed signs of osteomyelitis [23]. Antimicrobial stewardship activities, including the tracking and monitoring of antimicrobial resistance and drug usage [24], contribute to unnecessary antibiotic overuse and low clinical failure.

A limitation of this study is that it was a single-center retrospective study. The sample size was not large enough to verify the association of some variables with certain outcomes. However, our study was well-organized and the data were collected coherently, which could overcome these limitations. In addition, medical documentation, compiled by a multidisciplinary team, underwent a comprehensive review, and 2 orthopedic physicians meticulously examined the surgical photographs to evaluate the presence of RI.

## CONCLUSIONS

The common causative organisms of DFO requiring amputation are *S aureus* and *Pseudomonas* spp. PAD (ABI <0.6) and hemodialysis were associated with poor clinical outcomes despite radical resection of the infected bone. As it is difficult to determine the extent of infection remaining after DFO amputation, it is necessary to consider the risk factors of additional amputation. Vigilant monitoring after amputation and antimicrobial stewardship based on microbiological epidemiology, prognostic factors, and the type of surgery, are important. A multidisciplinary team could assist in these activities to ensure treatment success.

## Supplementary Material

ofae236_Supplementary_Data

## Supplementary Data

Supplementary materials are available at *Open Forum Infectious Diseases* online. Consisting of data provided by the authors to benefit the reader, the posted materials are not copyedited and are the sole responsibility of the authors, so questions or comments should be addressed to the corresponding author.

## Notes

***Author contributions.*** Y. J. B. conceptualized the research question, conducted the analysis, and drafted the initial manuscript. J. J., S. H. W., S. Y. P., D. C., and T. H. K. provided thorough feedback on the research design and analysis. C. Y. A., E. M. K., and S. L. B. collected and analyzed the data. E. L. contributed to conceiving the ideas, supervising the study, and reviewing the manuscript. All authors read and approved the final manuscript.

***Acknowledgments.*** We thank all specialists of the DFI multidisciplinary team for sincerely treating patients with DFI to improve patient care and healthcare outcomes.

***Patient consent.*** The study protocol was approved by the ethical review committee of Soonchunhyang Hospital (2023-3-009). Obtaining informed consent from the patients was waived owing to the retrospective nature of the study.

***Data availability.*** The datasets used and/or analyzed during the current study are available from the corresponding author upon reasonable request.

***Financial support.*** This research was funded by Soonchunhyang University (No. 2024). This work was supported by the National Research Foundation of Korea grant funded by the Korea government (No. RS-2023-00219563).
