# Supplementary material for: Diabetic Foot Osteomyelitis Undergoing Amputation: Epidemiology and Prognostic Factors for Treatment Failure
Source: Open Forum Infect Dis. 2024 Jul 9;11(7):ofae236. doi: 10.1093/ofid/ofae236 (PMC11232692; doi:10.1093/ofid/ofae236)
Supplement: ofae236_Supplementary_Data [file ofae236_supplementary_data.docx]

**Additional File 1.** Factors associated with poor outcomes in patients with diabetic foot osteomyelitis requiring amputation.

|  | Crude OR (95% CI) | Adjusted OR (95% CI) | *p* |
| --- | --- | --- | --- |
| Residual infection | 2.57 (1.04–6.53) | 2.96 (0.94–9.87) | 0.06 |
| ABI |  |  |  |
| >0.8 | 1 | 1 |  |
| 0.6–0.79 | 2.29 (0.57–8.87) | 2.14 (0.48–9.29) | 0.31 |
| 0.4–0.59 | 9.60 (2.37–50.1) | 15.3 (3.10–102.7) | 0.002 |
| <0.39 | 2.95 (1.04–8.73) | 4.48 (1.34–16.4) | 0.018 |
| Haemodialysis | 2.26 (0.99–5.38) | 2.32 (0.81–7.24) | 0.13 |
| Dementia | 0.33 (0.05–1.42) | 0.39 (0.04–2.58) | 0.37 |
| Major amputation | 0.41 (0.15–1.05) | 0.81 (0.14–3.86) | 0.80 |
| Infected foot region |  |  |  |
| Forefoot | 1 | 1 |  |
| Midfoot | 0.65 (0.21–1.88) | 0.35 (0.06–1.64) | 0.20 |
| Hindfoot | 0.72 (0.20–2.32) | 0.60 (0.08–4.77) | 0.62 |

Abbreviations: ABI, ankle brachial index; OR, odds ratio.

**Additional File 2.** Additional outcomes of the four groups in this cohort study

|  | PC-GO  (n = 49) | PC-PO  (n = 26) | RI-GO  (n = 11) | RI-PO  (n = 15) |
| --- | --- | --- | --- | --- |
| Amputation | 0 | 14 (53.8) | 0 | 14 (93.3) |
| Antibiotics duration, median [IQR] | 15 [8–26] | 29.5 [18.5–48.5] | 44 [17–58.5] | 50 [23–62] |
| CDI | 5 (10.2) | 4 (15.4) | 3 (27.3) | 3 (20) |
| MDRO | 5 (10.2) | 5 (19.2) | 1 (9.1) | 2 (13.3) |
| In-hospital mortality | 1 (3.7) | 7 (33.3) | 3 (42.9) | 0 (0) |

Data are presented as number (%) unless otherwise described.

Abbreviations: IQR, interquartile range; CDI, *Clostridioides difficile* infection; MDRO, multidrug-resistant organism
